# Cardiac tamponade occurred after endoscopic submucosal dissection: conservative management of the esophagopericardial fistula

**DOI:** 10.1002/ccr3.1218

**Published:** 2017-10-20

**Authors:** Davide Bona, Greta Isabella Saino, Massimo Medda, Valerio Panizzo, Giancarlo Micheletto

**Affiliations:** ^1^ Laparoscopic Unit General Surgery Istituto Clinico Sant'Ambrogio University of Milan Milan Italy; ^2^ Laparoscopic Unit Istituto Clinico Sant'Ambrogio Milan Italy; ^3^ Interventional Cardiology Unit Istituto Clinico Sant'Ambrogio Milan Italy

**Keywords:** Cardiac tamponade, endoscopic submucosal dissection, esophageal squamous cell carcinoma, esophagopericardial fistula, stent‐in‐stent procedure

## Abstract

We describe the case of an esophagopericardial fistula generated after endoscopic submucosal dissection in a patient affected by a superficial esophageal squamous cell carcinoma immediately treated with percutaneous pericardial drainage and placement of a partially covered self‐expanding metal stent that has been removed using the stent‐in‐stent technique after 35 days.

## Introduction

The improvement of endoscopic imaging increased the number of the esophageal superficial tumors detected. Endoscopic submucosal dissection (ESD) is now widely considered a valid approach in the treatment of superficial squamous cell carcinoma of the esophagus (ESCC). ESD allows an en bloc resection of lesions greater than 20 mm in diameter and provides a reduction in the recurrence rate compared to endoscopic mucosal resection (EMR) 1. ESD is technically more difficult compared to EMR, and also it has a major complication rate. Esophageal perforation is the major complication with an incidence of 0–6.9%, and it is burdened with a high mortality rate of 11.9% 1, 2, 3. Intraoperative detection and treatment of perforation reduce the mortality rate and prevent a surgical treatment. Conservative management of iatrogenic esophageal fistulas after ESD is nowadays considered the first choice of treatment 4. Currently, the endoscopic techniques that provide the best exclusion of an esophageal fistula are the use of self‐expanding metal stents (SEMSs) or the use of an over‐the‐scope‐clipping system (OTSC System—OVESCO Endoscopy AG). In our experience, we use OTSC System when there is a small estimated defect with a maximum diameter of 1 cm and when the margins are not devitalized 5; if there is evidence of a larger defect (>1 cm), the best solution is to place a SEMSs.

## Case Report

**Figure 1:**
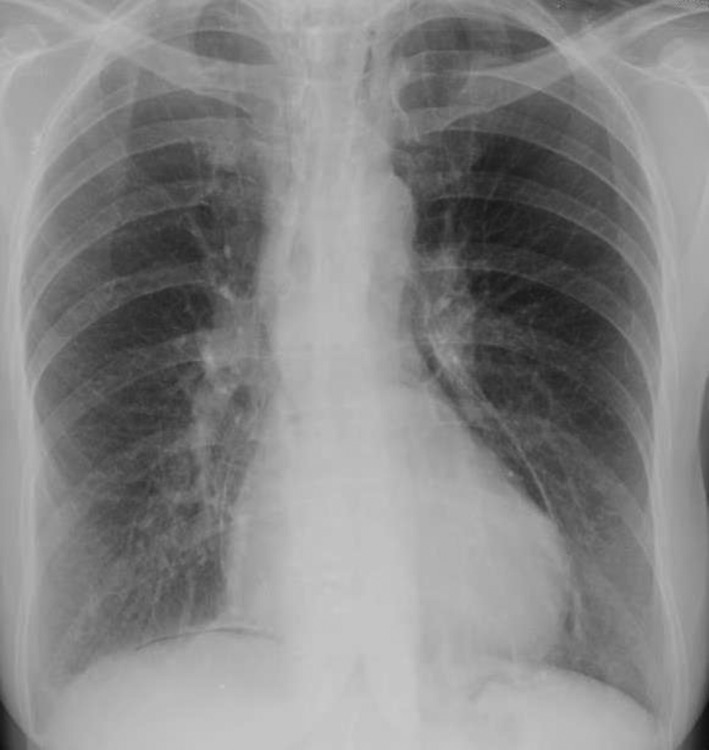
Chest x‐ray shows the presence of pneumopericardium and a thin right subphrenic sickle air without evidence of pneumothorax.

**Figure 2:**
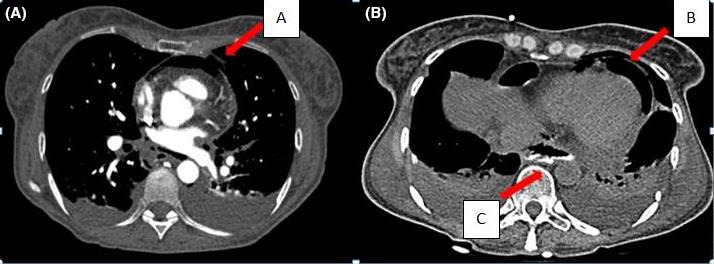
Chest CT scan with intravenous (A) and oral contrast medium (B) shows an idropneumopericardium (A, B arrows), pneumomediastinum, esophagopericardial fistula (C arrow), and bilateral pleural effusion.

**Figure 3:**
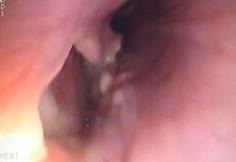
Endoscopic view of the full thickness esophageal fistula that involves 1/4 of the circumference on the left side of the esophagus.

During an endoscopy for a suspected GERD, a 56‐year‐old woman was diagnosed a plane, nonulcerated, slightly depressed esophageal lesion of the middle thoracic esophagus located at 33 cm from the mouth. This lesion was classified as a Paris type 0‐IIc with a diameter of 20 mm. Biopsies showed a G1 squamous cell carcinoma. The clinical staging of the lesion based on total body CT scan and EUS highlighted no submucosal (m1) and no lymph nodes involvement—cT1aN0M0. The patient's past history was otherwise negative. After a multidisciplinary consulting, we decided to treat the neoplasia with ESD; the patient was consenting, and we took the informed consent. The procedure was performed under general anesthesia, in supine position, using a single‐channel upper gastrointestinal endoscope; the perimeter of the lesion was marked using argon plasma coagulation (APC) spots, and we proceed with the submucosal injection of 0.4% sodium hyaluronate (MucoUp^®^; Johnson and Johnson, Tokyo, Japan) diluted with normal saline solution to create a submucosal lifting. The lesion was on the left side of the esophagus end, and the dissection was performed with a step‐by‐step electrocauterization using HookKnife (Olympus America) and positioning a transparent cap on the tip of the endoscope to keep the resected flap of mucosa out of the endoscope view. Dissection time was 75 min. At the end of dissection, there was no evidence of subcutaneous emphysema. Three rotatable endoscopic clips (Quick Clip 2—Olympus) were applied to close the mucosal gap. There have been no salient problems during the first postoperative day, and a water‐soluble contrast X‐ray of the esophagus showed a regular transit without extraluminal presence of contrast medium. Two days later, 72 h far from the dissection, the patient started with fluid diet and while she was drinking water she had a strong retrosternal pain. An urgent ECG showed a ST elevation in leads II, aVF, and V4‐5‐6. The chest X‐ray was indicative of the presence of a pneumopericardium (Fig. 1). The clinical course deteriorated rapidly with the appearance of worsening hypotension and tachycardia. An urgent thorax CT scan with intravenous and oral contrast medium showed an idropneumopericardium conditioning a cardiac tamponade with associated pneumomediastinum, reactive bilateral pleural effusion, and evidence of esophagopericardial fistula (EPF) (Fig. 2). The patient was immediately submitted to a percutaneous pericardial drainage with an outflow of 60 cc corpuscolated pericardial effusion and 250 cc underpressure air; the critical clinical condition was rapidly improved. The endoscopy showed a mucosal interruption due to the fall of the proximal clip. We preferred to remove the other two clips and so we identified a full thickness solution in the esophageal wall and we decide to put a self‐expanding partially covered metal stent (SEMSs) to exclude the fistula from salivary and alimentary transit (Fig. 3). We placed an Ultraflex proximal release 23 × 18 cm (Boston Scientific, Marlborough, MA) and a naso‐jejunal tube for enteral nutrition. The procedure was completed by the positioning of two percutaneous pleural drainages. The patient was referred to an intensive care unit and started parenteral nutrition and antibiotic therapy (metronidazole 500 mg bid, linezolid 600 mg bid, and fluconazole 200 mg die). Three days after the stent placement, we proceeded with a radiologic study of the esophagus using water‐soluble contrast and there was no evidence of extraluminal spreading and also no evidence of periprosthetic leak so the patient started with a soft diet and she was discharged from hospital 6 days later. The pericardial drainage was kept in place 4 days to obtain a complete resolution of air outflow and the appearance of pericardial clear serum. We did not perform pericardial antiseptic washes through the pig‐tail. In our experience, we are used to leave the stent in place at least 30–40 days because the average time of healing of the esophageal fistulas is 3–4 weeks, as described in literature 6. The endoscopic evaluation after 35 days showed a complete epithelialization of proximal and distal uncovered part of the stent, and it was not possible to remove the device. According to the stent‐in‐stent procedure, we positioned a second self‐expanding fully covered stent of the same size and length, in order to obtain the necrosis of the tissue between the stents induced by compression and easily remove the stent few days later 7, 8. The stent‐in‐stent positioning was performed under radioscopic control using a proximal release stent. After 7 days, we rapidly removed the fully covered stent and we observed an almost complete necrosis of the ingrowth tissue in the distal part of the deep esophageal prosthesis but an incomplete visualization of the proximal one. We decide to remove the stent taking the highly visible green suture positioned at the distal end of the stent inducing a caudo‐cranial intussusception of the stent. The endoscopic evaluation after removal showed a complete closure of the esophageal wall defect. The next day, the patient started with a semisolid diet and she was discharged in 48 h later. The histologic report of the specimen showed a complete abscission of the esophageal lesion confirmed as a G1 squamous cell carcinoma—pT1a. An esophagogastroduodenoscopy performed 3 months after was negative for recurrence and esophageal stenosis.

## Discussion

Temporary stent placement can be considered for treating esophageal leaks, fistulas, and perforations, but the optimal stenting duration remains unclear and should be individualized according to personal experience. The European Society of Gastrointestinal Endoscopy (ESGE) recommends for the removal of embedded partially covered esophageal prosthesis the use of stent‐in‐stent technique (strong recommendation, low quality of evidence) 9. The choice of a large and partially covered stent is due to the fact that in our experience, the wide proximal calyx (28 mm of diameter) and the absence of external covering allow a rapid reimbursement of the stent to the esophageal wall; the compression of the mucosa facilitates the growth of granulation tissue that stabilizes the stent avoiding the distal migration. However, we know that the removal of this type of stent could be difficult. The use of covered self‐expandable metal stents could also be considered as a good choice of treatment of esophageal fistulas and leaks 10. The use of fully covered stents is however burdened by a greater incidence of distal migration and periprosthetic endoleak. On the other hand, the removal of covered stents is easier and less dangerous.

In our experience, 13 consecutive patients underwent conservative treatment of esophageal fistula using partially covered SEMSs (Boston Scientific) (*n* = 6) or over‐the‐scope‐clipping system (OTSC System—OVESCO Endoscopy AG) (*n* = 7). For small defects with a maximum diameter of 1 cm, we used OTSC System, but when there was evidence of a larger defect (>1 cm), we preferred to place a SEMSs 5. We have treated six patients with the stent‐in‐stent technique. The first SEMSs remained in place for a median of 39 days (range 18–68) without displacement. All stents were left in place for a median of 9 days. The overall stent‐in‐stent success rate was 100% for the removal of embedded stents. No serious adverse events related to the procedure occurred. We observed a 100% success rate in the closure of the fistula. We consider the procedure safe, well tolerated, and effective. The use of a covered Ultraflex stent of the same size as the old stent for a limited time (<8 days) was consistently successful. In case of ineffective liberation of the proximal calyx of the stent, its removal for caudo‐cranial intussusception is possible and sometimes essential. We used this maneuver twice, and we consider that it allows to avoid a longitudinal traction on the proximal part of the stent facilitating a tissue detachment from the prosthesis exerting a centripetal force of much lower magnitude. Conservative management of EPF occurred during ESD and treated with esophageal stenting and pericardial drainage is feasible and safe. The use of partially covered stent has to be reserved at the time in specialized centers because of possible technical difficulties during the removal.

## Conflict of Interest

None declared.

## Authorship

DB: wrote the manuscript and managed the patient. GIS: wrote the manuscript and managed the patient. MM: performed the pericardial drainage. VP: managed the patient. GM: managed the patient.
